# Assessing genetic conservation of human sociability-linked genes in *C. elegans*

**DOI:** 10.1007/s10519-025-10216-2

**Published:** 2025-02-21

**Authors:** Mila C. Roozen, Martien J. H. Kas

**Affiliations:** Groningen Institute for Evolutionary Life Sciences, University of Groningen, Groningen, The Netherlands

**Keywords:** Social behavior, Evolution, Genetic conservation, *Caenorhabditis elegans*, *ACVR2A*

## Abstract

Social behavior is a common though variable trait across animal species. How much of the variation in social behavior is due to biological common mechanisms across animal species is unknown. In this study we examined to what extent human genetic variation in sociability is affected by pathways shared with *Caenorhabditis elegans* and whether any conserved sociability-linked genes show enhanced levels of essential functions and interactivity. We found inconsistent evidence of increased conservation with more thorough analyses resulting in no evidence of increased conservation of human sociability-linked genes. Conserved genes were highly interactive compared to nonconserved and random genes, while only a limited number of genetic interactions were found to be conserved. No evidence was found for enrichment of social phenotypes in *C. elegans* orthologs of human sociability-linked genes while evidence for associations with essential functions were limited. The activin A receptor type 2A (*ACVR2A*) gene appears to play a role in social behavior in both humans and *C. elegans*, making it an interesting gene for further study.

**Supplementary Information:**

The online version contains supplementary material available at 10.1007/s10519-025-10216-2.

## Introduction

Social or collective behavior is a trait observed across many species, from collective behaviors in single-celled slime molds (Reid and Latty 2016) to the complex social behaviors observed in primates and (by extension) humans (Roberts and Roberts 2016). Some aspects of social behavior serve functions with obvious benefits to reproductive fitness, such as finding and courting potential mates and caring for children in order to ensure survival of offspring. However, several species also engage in social behaviors with less obvious effects on reproductive fitness or that may even seem detrimental. For example, in the nematode *Caenorhabditis elegans* (*C. elegans*), chemical signaling between individuals can result in a reduced lifespan (Ludewig et al. 2017). As another example, bonobos, the closest living relatives to humans, share food with strangers even when there is no apparent benefit to themselves (Tan and Hare 2013).

Although social behavior is evident in many species, the quantity of social contact engaged in by individuals from different species and the complexity of the social structures created by those individuals vary to a strong degree. Some species are mostly solitary and engage in social behavior mostly when it directly increases fitness, such as for feeding, mating or parenting (for example pumas (Elbroch et al. 2017) and koalas (Ellis et al. 2009)) while some other species spend most or all of their lives in social groups, such as chimpanzees and bonobos (Gruber and Clay 2016) as well as several species of eusocial insects (Robinson et al. 1997).

The consistent variability in social behavior between species indicates that genetic variation in social behavior exists and has been under natural selection across speciation. In primates, which display strong variations in the extent to which they engage in social behaviors with kin and strange animals, interspecies variation in social behavior has been linked to variation in brain size, leading to the hypothesis that the large brain sizes observed in some primates (such as humans) are the result of long-term selection on social complexity (the ability to cognitively handle high numbers of complex social relationships) (Dunbar 2009).

On the other hand, the fact that social behavior is common across species indicates some shared genetic basis across species. Evidence for such a shared basis of social behavior between humans and other species has been provided by experimental studies using genetic manipulations in model animals. Genes associated with extreme variations in social behavior in humans can be altered in model animals in order to examine whether these genes affect social behavior in these animals in a similar manner. For example, a gene known to be involved in the extreme sociability of individuals with Williams Syndrome (a deletion on a section of chromosome 7), *GTF2I*, similarly increases social behavior towards strangers in mice when gene function is disrupted (Sakurai et al. 2011) and structural gene variants in this gene are associated with the stereotypical hypersociability seen in domesticated dogs (vonHoldt et al. 2017). Several successful mouse models (such as *Shank3* and *Foxp2*) have been created based on human mutations associated with autism spectrum disorders, which are characterized in part by aberrant social behavior (Crawley 2012).

Although cross-species studies have elucidated important aspects of the evolution of social behavior across species, much is still unknown about the evolution of the molecular substrates underlying such behavior. While many species display some form of social behavior at the very least in the pursuit of procreation, this does not necessarily mean that the genetic background underlying such behavior is similar between species, especially those far removed from each other. However, three recent studies have provided some evidence that might point to the conservation of molecular mechanisms underlying social behavior across far removed species. In three separates studies, Kasap et al. (2018), Franklin and Dwyer (2021) and Sall et al. (2021) found evidence that putative risk genes for schizophrenia, bipolar disorder and major depressive disorder were highly conserved between *C. elegans* and humans, indicating that the molecular basis for these disorders may have been present even in the last common ancestor of humans and *C. elegans*. In these studies, they found that these genes were associated with essential functions for reproductive fitness (survival and reproduction) and had heightened levels of interactivity, which may both explain why these genes are so highly conserved.

These findings could indicate that similar processes have affected genes related to social behavior, as these disorders are commonly associated with social dysfunction (Grant et al. 2001; Lahera et al. 2015; Kupferberg et al. 2016; Tatay-Manteiga et al. 2018; Saris et al. 2022) and genetic studies hint at shared molecular substrates (Bralten et al. 2021; Álvaro Andreu-Bernabeu et al. 2022). However, it might also be the case that the presence of enhanced genetic conservation found for the three neuropsychiatric disorders is due to some other shared factors across the disorders and unrelated to social behavior, or perhaps it is a characteristic of neuropsychiatric disorders specifically. In this case, genes for which variability is associated with differences in sociability might be conserved similarly to the total protein coding genome. Another possibility is that conservation of social behavior is low, because the behavior itself varies strongly between humans and *C. elegans*.

Studies of *C. elegans* behavior have found several behaviors which can be described as social. While most *C. elegans* are hermaphrodites with the ability to self-fertilize, a minority is male, leading to the presence of mating behavior. Male *C. elegans* rely on chemical signals such as ascaroside pheromones to find optimal mates (Luo et al. 2024). *C. elegans* nematodes with a specific mutation in the npr-1 gene have been known to engage in social feeding, in which the nematodes aggregate around the border of the bacterial lawn which serves as food. It has been found that this behavior is partially driven by oxygen tension preferences (Gray et al. 2004). However, it has been demonstrated that ascaroside pheromones also play a role in the aggregation behavior, indicating that there is a social component to the aggregation behavior (Macosko et al. 2009). Ascaroside pheromones have also been found to communicate other signals to other individuals as mentioned above, such as signals affecting developmental progression and life span (Ludewig et al. 2017) or signals attracting other individuals resulting in aggregation (Srinivasan et al. 2012). The neuropeptide nematocin, homologue of the mammalian peptide hormone oxytocin, may be involved in signals from *C. elegans* larvae affecting the feeding behavior of adult nematodes (Scott et al. 2017). These and other studies show that *C. elegans* has a wide repertoire of social behaviors, which could potentially share some genetic background with human social behavior.

In this study, we will examine to what extent human protein-coding genes with sequence variation associated with human variability in sociability are conserved between humans and *C. elegans*. We expect conservation to be heightened for sociability-linked genes compared to the total human protein-coding genome, similar to the findings for neuropsychiatric disorders, and will test this hypothesis of hyper conservation against the null hypothesis that conservation is similar for genes associated with human sociability compared to the total protein coding genome. We will examine whether the social function of any genetically conserved genes is conserved to determine whether any conserved genes could potentially constitute a shared genetic basis for sociability between humans and *C. elegans.* Finally, we will examine whether conserved genes are enriched for lethal and sterile phenotypes in *C. elegans* and whether conserved genes show enhanced interactivity compared to non-conserved sociability-linked genes to examine potential causes of the long-term conservation.

## Method

### Data collection

Seventy-six genes associated with genetic variability potentially related to sociability were extracted from the sociability GWAS performed by Bralten et al. (2021). We will use the term ‘sociability-linked genes’ for these genes in this paper, however it is important to keep in mind that while polymorphisms in these genes might affect sociability, this does not mean that sociability is a main function of these genes. Seven of these were excluded from further analysis, either because they were not included in the current Ensembl (Cunningham et al. 2022) database (3 genes) or because they did not code for any proteins, indicating they could not be analyzed using the methods planned for this study. This resulted in a total of 69 sociability-linked genes which were included in the following analyses.

### Ortholog detection

A stepwise approach was taken to determine whether genes were conserved. The first step was to search for established orthologues in the Ensembl database (Cunningham et al. 2022). The second step was to search for established orthologs in the WormBase database (WormBase version WS285, http://www.wormbase.org; Davis et al. 2022). Finally, if no ortholog was found in the first steps, BLASTP was used (in WormBase) to determine whether the largest transcript of each gene had a clear counterpart in *C. elegans*. The total overview of the genes with their respective orthologues and the method through they were discovered, see Supplementary Table 1.

In order to determine whether BLASTP results of similar proteins could indeed be considered counterparts, BLASTP hits were evaluated against criteria based on those by Kasap et al. (2018) and based on the discussion by Pearson (2013). Transcripts were considered counterparts if (1) they did not differ over 100 amino acids in length; (2) the E-value of the hit was below 10^–4^; (3) the identity was at least 20% for a segment of at least 50 amino acids in length and (4) for at least three species with established orthologs of the human gene, the same *C. elegans* gene was also a hit using BLASTP.

### Statistical analyses

All analyses were carried out in R using RStudio (R version 4.2.1, RStudio version 2022.07.2). The number of orthologs were compared with data from previous efforts to study the level of conservation found across the human genome. In a large effort to determine orthologs between human and *C. elegans* genes, Kim et al. (2018) found that approximately 52.6% (10,678 out of 20,310 genes) of the human protein-coding genome had recognized orthologs in *C. elegans* at that time. We used Fisher’s exact test to examine whether the differences between the proportion of human genes with *C. elegans* orthologs according to Ortholist II (Kim et al. 2018) and the proportion of human protein-coding sociability-linked genes with a *C. elegans* ortholog. The genes from the sociability set were removed from the total human gene set to create independent gene sets.

However, as the determination of homologs in the study by Kim et al. (2018) was not directly comparable to the methods used in this study, we also compared the sociability set to ten random sets of human genes created using the Molbiotools Random Sequence Generator (https://molbiotools.com/randomsequencegenerator.php) with the same number of genes as the sociability set. The random human gene sets created for the analysis of the level conservation and the interactivity can be found in Supplementary Table 2. The comparisons were planned to be carried out using chi-squared tests unless any expected counts in the contingency table are below 10, in which case Fisher’s exact test will be utilized. In addition, the z-score of the sociability set was compared to the distribution of all sets (random and sociability) to determine whether the sociability set is an outlier.

The function of conserved genes was examined in *C. elegans* using the WormBase Worm Phenotype Ontology database (Schindelman et al. 2011) to determine whether (a) conserved sociability-linked genes constitute a shared genetic basis for sociability between humans and *C. elegans* and (b) whether conserved sociability-linked genes may have been conserved as a result of functions essential to fitness. As described in the introduction, one type of social behavior which has been previously studied in *C. elegans* is social feeding. Social feeding can be measured by the aggregation of the nematodes or by ‘bordering’, a behavior typical of social feeding animals whereby the animals feed from the edges of a bacterial lawn (the food source). The WormBase Phenotype Ontology dataset includes four phenotypes related to social feeding: social feeding, solitary feeding, bordering and non-bordering. We also examined three phenotypes related to pheromone communication: pheromone production, pheromone sensation and pheromone behavioral response. We used WormMine (http://intermine.wormbase.org/tools/wormmine/begin.do) to query the *C. elegans* genes in the WormBase dataset for these phenotypes and we examined the presence of social phenotypes in *C. elegans* orthologs of human sociability-linked genes to determine whether these genes constitute a shared genetic basis of sociability.

In order to determine whether human sociability-linked genes were conserved between humans and *C. elegans* as a result of essential functions, we repeated the above analysis but instead queried WormBase for the five phenotypes related to fitness: ‘lethal’, ‘embryonic lethal’, ‘larval lethal’, ‘sterile’ and ‘sterile progeny’. For each phenotype, a subset was retrieved from the data for *C. elegans* orthologs of human sociability-linked genes which were associated with the phenotype. We then compared the proportion of each of these phenotypes in the orthologues of the human sociability-linked genes with the proportion of these phenotypes in the total set of 19,985 *C. elegans* protein-coding genes available in the WormBase dataset (Davis et al. 2022) to determine whether the conserved genes are more likely to have any of these essential functions in *C. elegans*. The analyses were performed using chi-squared tests of independence if all expected cell counts were above 10 or Fisher’s exact test if this was not the case.

As a final step we wanted to examine whether certain sociability-linked genes might be conserved between *C. elegans* and humans as a result of having particularly high numbers of interactions with other genes. Interactions were examined using GeneMANIA (Wade-Farley et al. 2010). The ‘max resultant gene’ setting, which determines how many genes from outside the gene sets could be used to create interactions was set to 0, as well as the ‘max resultant attributes’. We created two human gene sets, one with the conserved sociability-linked genes and one with the non-conserved sociability-linked genes, in order to examine whether sociability-linked genes in general or conserved sociability-linked genes specifically showed increased levels of interactions. We compared the gene sets each to the ten random gene sets created for the comparison regarding the level of conservation, where the sets were split into two sets to conform to the sizes of the conserved and non-conserved sets. In order to perform a second check whether any potential increased interactivity between sociability-linked genes is a result of the genes being involved in the same phenotype, we performed the analysis a second time, changing the ‘max resultant gene’ setting to 20. In this manner, highly interactive genes from the random sets would be detected even if these interactions were found outside of the random genes with which they are assigned. Statistical tests were performed using ANOVA models. If assumptions of the ANOVA model are violated, generalized linear models with appropriate model specifications were used.

The Molbiotools Random Sequence generator does not include an option to create random sets for *C. elegans*. In order to be able to compare the interactions found in human sociability-linked genes with those found in the *C. elegans* orthologs, we downloaded the full set of *C. elegans* protein-coding genes from WormBase and sampled 10 random sets, each of the same size as the set of *C. elegans* sociability orthologs. The random gene sets can be found in Supplementary Table 3. In order to create an approximately fair comparison, we grouped the orthologs per human gene for which they were homologous, because *C. elegans* genes which were homologous to the same human gene were highly interactive amongst themselves. We then counted the interaction with other gene groups. In total, this led to 49 gene groups (one for each human gene minus one, as two human genes shared the same *C. elegans* ortholog). Statistical comparisons are carried out similar to those for human genes. Finally, we examined whether any interactions between genes were conserved between humans and *C. elegans.*

## Results

### Conservation

Thirty-five out of 69 human protein-coding sociability-linked genes (51%) had a registered *C. elegans* ortholog in the Ensembl database. The search for orthologs of human sociability-linked genes in *C. elegans* using WormBase led to the addition of 13 out of the remaining 35 genes, leading to a total of 48 out of 69 sociability-linked genes (70%) with a known ortholog. The search for counterparts of gene products using BLASTP led to the discovery of 2 orthologs in *C. elegans*, resulting in a total of 50 out of 69 (72%) conserved sociability-linked genes in *C. elegans*. Two human sociability-linked genes were orthologs of the same *C. elegans* gene. As a result of one-to-many and many-to-many orthologs, the list of 50 conserved human sociability-linked genes resulted in 70 *C. elegans* sociability orthologs, of which 4 were pseudogenes, which were not included in subsequent analyses, while the remaining 66 genes were protein coding genes. Based on the comparison with the Kim et al. (2018) data, the proportion of *C. elegans* orthologs appears significantly increased in the set of human sociability-linked genes compared to the total human protein-coding gene set (excluding the sociability-linked genes) (*X*^2^ = 10.20, *p* = 0.001). However, the comparison to the ten random sets showed zero pairwise differences between the sociability set and the random set (see Table 1 for the full results). The sociability set had a z-score of 0.49, indicating that there is no evidence that the sociability score deviates from the norm in regards to level of conservation. The random sets showed much higher percentages conserved genes compared to the Kim et al. (2018) data, likely because the method of determining which genes are conserved is more lenient, which could also account for the significant difference in the level of conservation of the sociability set as determined by our method compared to the level of conservation found by Kim et al. (2018).

**Table 1:** Pairwise chi-squared tests comparing gene conservation in random sets to conservation in the sociability set

Set	# Conserved (%)	*X*^2^	*p*-Value
1	44 (63.77%)	0.83	0.361
2	51 (73.91%)	0	1
3	53 (76.81%)	0.15	0.696
4	47 (68.12%)	0.14	0.710
5	41 (59.42%)	2.07	0.151
6	49 (71.01%)	0	1
7	50 (72.46%)	0	1
8	51 (73.91%)	0	1
9	44 (63.77%)	0.83	0.361
10	50 (72.46%)	0	1
Sociability	50 (72.46%)	–	–

Next we will discuss the results regarding the conservation of social function in *C. elegans* orthologs of human sociability-linked genes. None of the examined social phenotypes showed a significant increase in the *C. elegans* orthologs of human sociability-linked genes. In fact, only one of the *C. elegans* orthologs of the human sociability-linked genes, *daf-1* (which is an ortholog of the human gene *ACV2RA*), was associated with any of the seven social behavior phenotypes examined, specifically, *daf-1* had been found to be associated with social feeding. However, the WormBase catalogue showed a dearth of results for the social behavior phenotypes in general, with no results based on RNAi studies for solitary feeding, pheromone sensation, pheromone production and pheromone behavioral response and no results in any type of study for the non-bordering phenotype. Therefore, this result may simply point to a lack of research into social phenotypes in *C. elegans.*

The results of the analysis regarding the presence of essential phenotypes among *C. elegans* orthologs of human sociability-linked genes are covered in Table 2. Several *C. elegans* orthologs of human sociability-linked genes were associated with one of the essential phenotypes. All essential phenotypes had expected cell counts below ten, therefore all analysis were carried out using Fisher’s exact test. Using the full set of 66 protein-coding orthologs, none of the phenotypes showed enrichment in orthologs of human sociability-linked genes.

**Table 2:** Enrichment of essential phenotypes in *C. elegans* orthologs of human sociability-linked genes, including and excluding the *srsx* gene family

Includes srsx gene family	Phenotype	Proportion in total protein-coding genome	Proportion in sociability orthologs	OR (95% CI)	*p*-Value
Yes	Lethal	0.09	0.17	0.51 [0.26–1.08]	0.507
	Embryonic lethal	0.15	0.21	0.66 [0.36–1.29]	0.168
	Larval lethal	0.04	0.05	0.90 [0.29–4.49	0753
	Sterile	0.12	0.18	0.62 [0.33–1.28]	0.131
	Sterile progeny	0.04	0.02	2.42 [0.42–97.11]	0.733
No	Lethal	0.09	0.19	0.42 [0.22–0.91]	0.018*
	Embryonic lethal	0.15	0.25	0.54 [0.29–1.08]	0.061
	Larval lethal	0.04	0.05	0.77 [0.25–3.86]	0.509
	Sterile	0.12	0.21	0.51 [0.27–1.08]	0.063
	Sterile progeny	0.04	0.02	2.08 [0.36–83.87]	0.724

*OR* odds ratio, *CI* confidence interval

*Significant at alpha = 0.05

One human sociability gene which may have affected the results of the analysis about essential phenotypes is *OR5B17*. WormBase indicates that this gene is an orthologue of a group of 13 genes from the *srsx* family, 9 of which are protein-coding. The *srsx* gene family is one of the families of highly divergent chemoreceptor gene families in *C. elegans* (Robertson and Thomas 2006). We therefore also carried out the analysis excluding the *srsx* genes which did not have any information available, removing all *srsx* genes from the data. This does remove one gene with lethal and sterile phenotypes from the data (*srsx-39*), but it also removes 8 genes with no essential phenotypes. Although most of the results do not change based on this adjustment, the enrichment of lethal genes in *C. elegans* orthologs of human sociability-linked genes became significant (Odds ratio = 0.42, 95% CI 0.22–0.91, *p* = 0.018).

The random human gene sets created for the analysis of the level of interaction can be found in Supplementary Table 2. The 50 conserved sociability-linked genes were compared with ten sets of 50 random human genes each and the 19 non-conserved genes were similarly compared with ten sets of 19 random genes as the total possible number of interactions is constrained by the total number of genes, therefore comparing sets of 19 to sets of 50 genes would lead to biased results.

Levene’s test for equality of variances showed that for both the conserved (F = 12.12, *p* < 0.001) and non-conserved (F = 2.98, *p* = 0.002) groups of sets, the sets varied significantly in their variances. As a result, generalized linear models using Poisson distributions were considered. However, significant overdispersion (difference between mean and variance) was observed for the conserved set and its comparison sets (dispersion = 1.76, *p* < 0.001), which violates the assumptions of the Poisson model. This was not the case for the non-conserved set and its comparison sets (dispersion = 1.09, *p* = 0.151). Instead, a quasipoisson model was used which estimates the variance instead of assuming equal mean and variance. This method will be used for the non-conserved set and associated random sets as well.

**Fig. 1:**
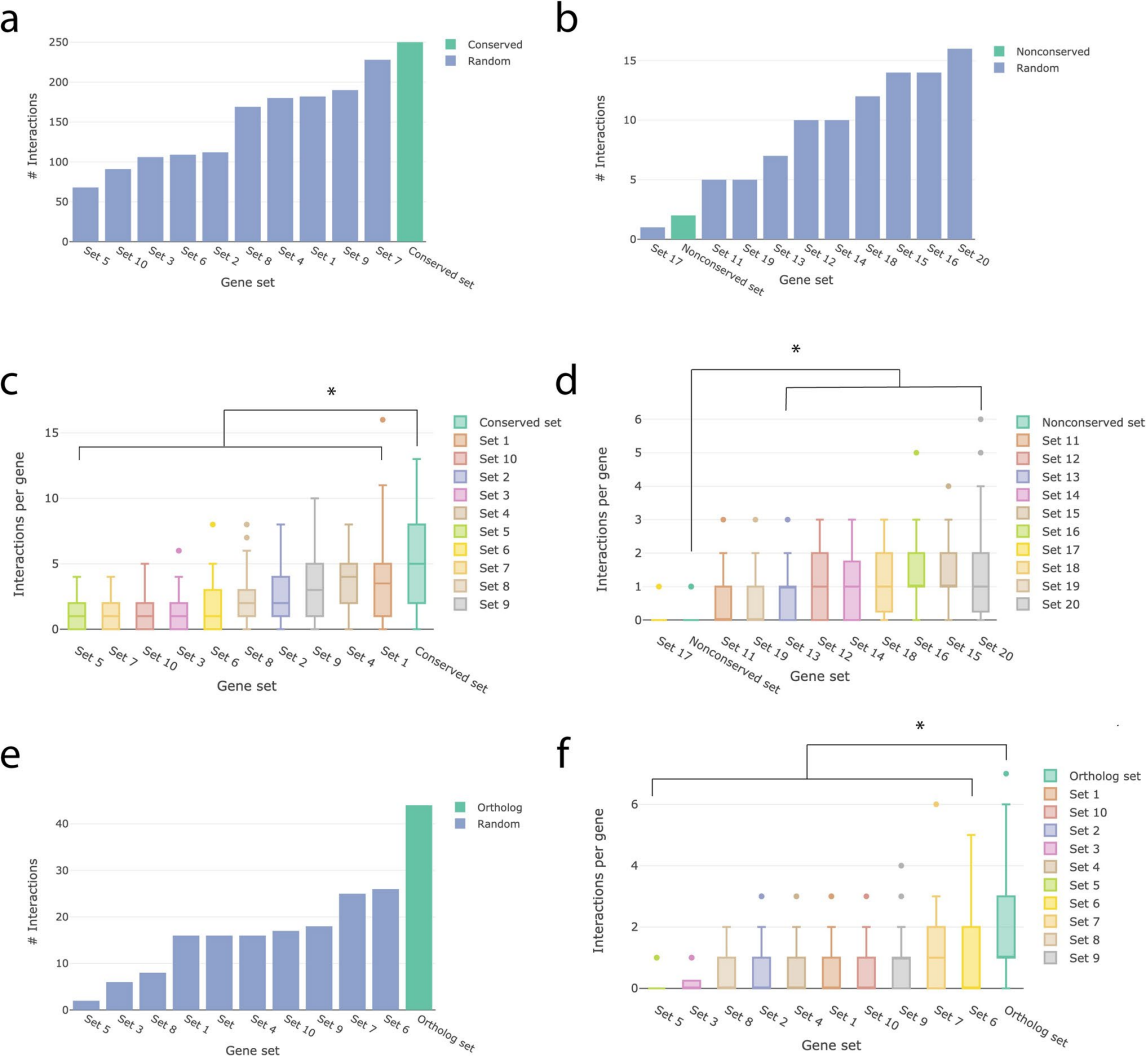
Interaction analyses showed increased interactivity between human sociability-linked genes conserved between humans and *C. elegans*. **a** Total interactions between conserved sociability-linked genes and random sets of 50 human genes; **b** Total interactions between non-conserved sociability-linked genes and random sets of 19 human genes; **c** Interactions per gene for conserved sociability-linked genes and random sets of 50 human genes; **d** Interactions per gene for non-conserved sociability-linked genes and random sets of 19 human genes; **e** Total interactions between *C. elegans* orthologs of human sociability-linked genes and random sets of 49 *C. elegans* genes; **f** Interactions per gene for *C. elegans* orthologs of human sociability-linked genes and random sets of 49 *C. elegans* genes. *p < 0.05

**Fig. 2:**
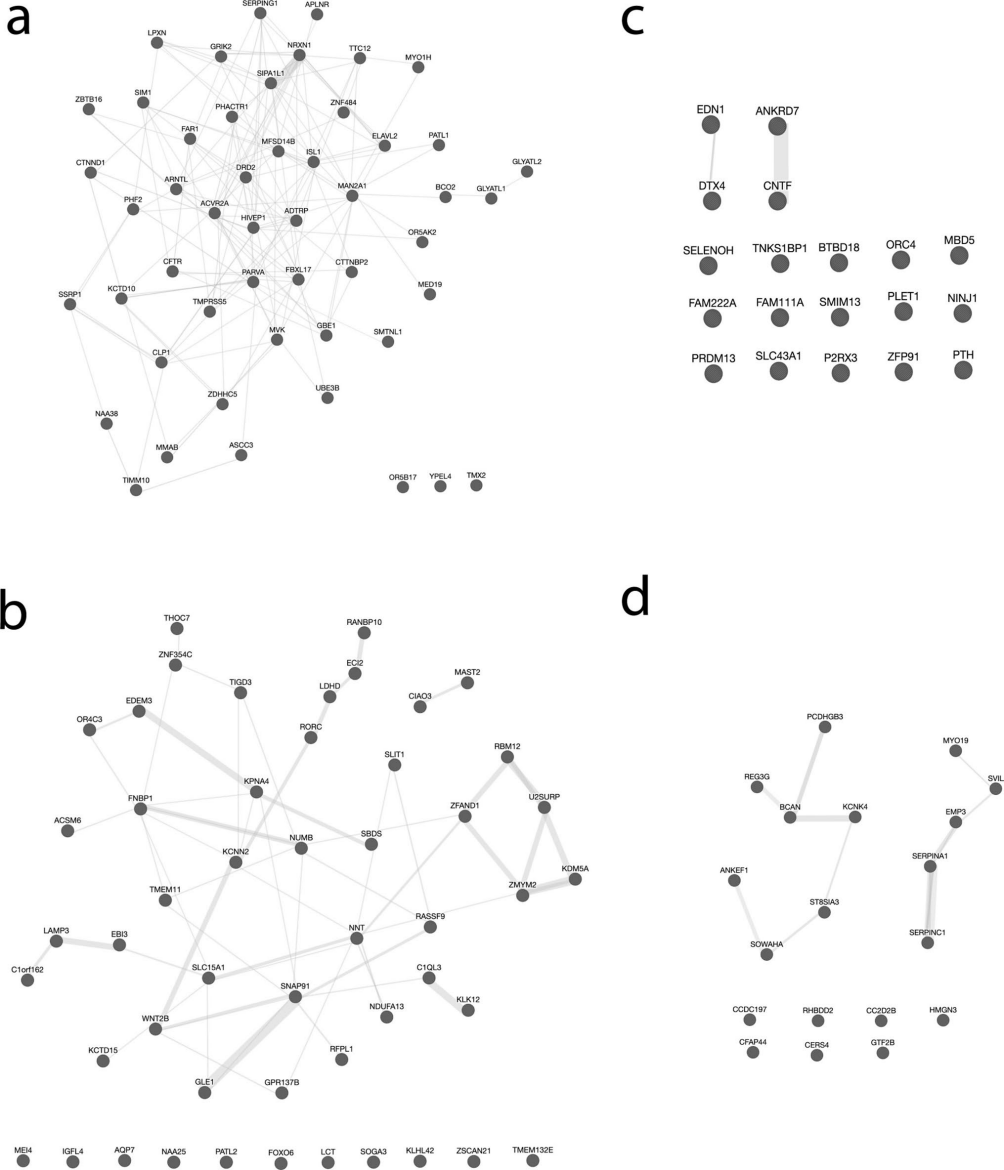
Network images from sociability-linked gene sets (**a** for conserved genes and **c** for non-conserved genes) and random gene sets of the same size (**b** and **d**) as the conserved (**a**) and non-conserved (**c**) sociability-linked gene sets. Random sets 8 (**b**) and 12 (**d**) were used for visual comparison as their total number of interactions was the mode across all gene sets of their respective sizes

The conserved set had significantly more interactions between genes compared to each random set separately (Figs. 1a–d and 2). The non-conserved set had the fewest total number of interactions and significantly fewer interactions compared to 7 of the random sets. In *C. elegans,* orthologs of human social complexity were significantly more interactive compared to random gene sets (Fig. 1e and f). Allowing for the inclusion of maximum 20 genes outside the gene set did not change the results in a significant way, the conserved set was still significantly more interactive compared to all associated random sets while the non-conserved set was now significantly less interactive compared to 5 of the associated random sets (Supplementary Fig. 1). Very few of the interactions found in the human data were also found in the *C. elegans* data. Only 9 out of 133 human sociability gene interactions were also found in *C. elegans* orthologs of these genes, whereas for *C. elegans ortholog gene interactions* this was 9 out of 44. The interactions found across species are displayed in Table 3.

**Table 3:** Interactions discovered both between human sociability-linked genes and between their orthologs in *C. elegans*

*Human gene 1*	*Human gene 2*	*C. elegans gene 1*	*C. elegans gene 2*
*ACVR2A*	*GBE1*	*daf-1*	*T04A8.7*
*APLNR*	*DRD2*	*npr-33*	*ser-5*
*ARNTL*	*SIM1*	*aha-1*	*hif-1*
*ARNTL*	*SIPA1L1*	*aha-1*	*F53A10.2*
*FBXL17*	*UBE3B*	*fbxl-1*	*oxi-1*
*GRIK2*	*NRXN1*	*glr-1*	*nrx-1*
*ISL-1*	*LPXN*	*lim-7*	*pxl-1*
*PARVA*	*SMNTL*	*pat-6*	*T15B12.1*
*PHF2*	*SSRP1*	*[jmjd-1.1 & jmjd-1.2]*^a^	*athp-1*

Square brackets indicate that this human gene has several *C. elegans* orthologs

^a^*jmjd-1.1* and *jmjd-1.2* are both orthologs of *PHF2*

## Discussion

In this study, we examined to what extent genes associated with variation in human sociability are conserved between humans and *C. elegans*. Using two different analyses, we found evidence both in favor of and in contradiction with the expectation that human sociability-linked genes would show heightened conservation between humans and *C. elegans*. One potential explanation for the result disagreeing with the hypothesis might be the methodological variation between this study and the studies previously performed regarding the conservation of genes associated with schizophrenia (Kasap et al. 2018), bipolar disorder (Franklin and Dwyer 2021) and major depressive disorder (Sall et al. 2021). While these studies searched for homologs using methods similar to those used in this study, their comparisons were similar to the one we carried out where the conservation in the sociability set was compared to that found in a previous study (Kim et al. 2018). However, as these studies used different methods from the one used by Kim et al. (2018) to determine homology, these comparisons may be biased and result in an increased likelihood to find significant results. The second method used in this study, utilizing random gene sets for the comparison may constitute a fairer comparison, and appears to indicate that conservation proportional to the total proteincoding genome is a more probable scenario than the hyper conservation found in the previous studies regarding neuropsychiatric disorders. These findings also highlight the importance in choice of methods for such studies, as suboptimal methods can lead to inaccurate results.

A similar explanation could explain the difference between our study and the studies mentioned above in the difference in essential phenotypes occurrence between gene sets and the total genome. Considering we used the same database to gather data for both the sociability set and the total protein-coding genome, this comparison may be considered more equal than comparing the data from WormBase to data from other studies. It may be the case that the enrichment for essential phenotypes found in the previous studies regarding the neuropsychiatric disorders might be reduced or disappear when they would be compared to the WormBase data. On the other hand, it could be the case that this difference underlies true variation in the nature of genes orthologous to human sociability-linked genes and those orthologous to human genes associated with neuropsychiatric disorders. It may be that genes associated with variation in the risk of neuropsychiatric disorders have larger effects on reproductive fitness compared to genes associated with reduced sociability, resulting in stronger constraints on the conservation of the former and therefore more likelihood that variation serves some essential function.

On the other hand, we did find evidence that human sociability-linked genes conserved between humans and *C. elegans* were more interactive both within the set as well as when taking into account interactions outside the set, compared to random human protein-coding genes and compared to non-conserved human sociability-linked genes, indicating that the increased interactivity was not a result of the fact that the genes were related to a single phenotype but specifically to the conserved nature of the genes. This finding has a seemingly logical explanation; highly interactive genes are more likely to be involved in important biological processes and therefore also more likely to be conserved (Brown and Jurisica 2007). However, this analysis is potentially biased as conserved genes are much more likely to be investigated in scientific research due to the potential translational value, therefore more interactions between conserved genes may have been discovered compared to nonconserved genes. As a result we cannot definitively conclude that conserved sociability-linked genes are more interactive than non-conserved genes. Future studies are required to determine whether conserved genes are indeed more interactive compared to nonconserved genes.

Our examination of the overlap in genetic interactions between human sociability-linked genes and the *C. elegans* orthologs of these genes appears to suggest that even when both human sociability-linked genes have orthologs in *C. elegans*, interactions found between the human sociability-linked genes are not often also found in their *C. elegans* orthologs. However, this interpretation is only valid if the gene interactions between the orthologs have actually been tested for in studies regarding protein interactions or gene co-expression. While the large databases of protein interactions and gene co-expression utilized by GeneMANIA and the large number of interactions between the *C. elegans* orthologs of human sociability-linked genes does suggest that it is likely these interactions have been tested for, to know for certain would require a thorough examination of the data or new studies regarding the protein interactions and co-expression patterns of *C. elegans* orthologs of human sociability genes. Therefore, while our data seems to suggest the interactions between human-sociability linked genes is not conserved in *C. elegans*, this conclusion requires further study to be confirmed.

One human gene which appears of particular interest based on our study is the activin A receptor type 2A (*ACVR2A*). This gene, which is conserved between humans and *C. elegans*, is the only gene which was found to have functions related to social behavior in both humans as well as in *C. elegans*. It was also found to be highly interactive (in fact, the most interactive gene among the conserved sociability-linked genes), interacting with 16 out of 49 (33%) of other conserved sociability-linked genes. Previous animal studies have demonstrated an important function of *ACVR2A* in regulating fertility and sexual behavior (Matzuk et al. 1995; Wreford et al. 2001; Ma et al. 2005). In *C. elegans*, the two orthologues of human *ACVR2A*, daf-1 and sma-6, have previously been implicated in egg laying behavior (Larsen et al. 1995), brood size (Maduzia et al. 2005) and sperm recruitment (McKnight et al. 2014), indicating that these orthologues affect reproductive fitness in *C. elegans* as well as in other model animals. In humans, *ACVR2A* is also known to be associated with human reproduction, for example through regulation of follicular development and oocyte maturation (Wang et al. 2022). These may indicate that the gene is conserved between humans and *C. elegans* as a result of very basic functions in reproduction, explaining its relevance to fitness.

While previous studies have demonstrated the conserved role of *ACVR2A* in fertility, the fact that *ACVR2A* may play a constitute a conserved genetic basis for sociability has not yet been established in previous research, potentially due to the relative novelty of the Bralten et al. (2021) GWAS study. The conservation of function of the *ACVR2A* gene between two such highly diverged species indicates the importance of this function for survival and reproduction across environments. This finding also highlights the potential of *C. elegans* for translational research, as studying the molecular processes affected by the *ACVR2A* gene in *C. elegans* may result in a better understanding of how such processes are involved in social behavior in humans (although the relation between *ACVR2A* and reproductive health may hamper the use of the orthologues of this gene as a good model), This may be especially useful in discovering the biological mechanisms underlying disordered social behavior, which is a common early symptom of several neuropsychiatric disorders such as major depressive disorder, schizophrenia and Alzheimer’s disease (Kas et al. 2019; Porcelli et al. 2019; Oliva et al. 2022) Looking at the finding from an evolutionary perspective, the discovery that *ACVR2A* may constitute a conserved biological basis for social behavior between humans and *C. elegans* paves the way for future research studying how genetic mechanisms have shaped social behavior across evolutionary time scales. Based on the high interactivity of the *ACVR2A* gene, it may also be interesting to examine how gene–gene interactions affect the function of the *ACVR2A* gene, both at a pathway level and at a behavioral level, to examine how intricate networks of genes can together affect complex phenotypes such as social behavior across species.

### Limitations

One important consideration for the drawing of conclusions from this study is that while the level of conservation of genetic sequences between species could indicate the level of functional conservation, it need not necessarily be so. While our results show no enrichment of conserved sociability-linked genes, it could still be the case that biological systems underlying social behavior have been disproportionally conserved between humans and *C. elegans* without it being reflected in the genome (or at least in the genes found by the GWAS study of Bralten et al. (2021)). For this study we were interested specifically in whether genetic sequences were highly conserved, however studies examining how biological functions may be involved in social behavior across species should look at those specifically. In fact, the involvement of the oxytocin counterpart nematocin found by Scott et al. (2017) is interesting in this regard due to the relevance of the oxytocin system in human social behavior (Froemke and Young 2021) and may constitute an interesting avenue for future research regarding the conservation of biological systems underlying social behavior between humans and *C. elegans*. In order to examine whether the genetic conservation found in our study corresponds to the conservation of biological function and how different biological pathways affected social behavior evolved across time, future studies could examine biological pathways associated with conserved and non-conserved human sociability-linked genes.

In this study, we combined three different sources of information to find orthologues for human genes with polymorphisms associated with sociability according to the GWAS by Bralten et al. (2021): Ensembl, Wormbase and BLASTP. We chose this combination of methods specifically to be able to compare our results to those from the previous studies regarding schizophrenia (Kasap et al. 2018), bipolar disorder (Franklin and Dwyer 2021) and major depressive disorder (Sall et al. 2021). However, the methods used by Ensembl (https://www.ensembl.org/info/genome/compara/homology_method.html) and Wormbase (https://parasite.wormbase.org/info/genome/compara/homology_method.html) to determine orthologues and the additional BLASTP method are not recent and novel methods have been proposed which are likely better at determining orthologues sequences (e.g. Malhis et al. 2019). While the chosen methods of this paper were considered sufficient to compare differences in the level of conservation between gene sets, it might be that the true level of conservation and the specific orthologues genes could be better determined using more advanced methods.

During our examination of social behavior phenotypes associated with the *C. elegans* orthologs of human sociability-linked genes it came to light that very few *C. elegans* genes have known associations with any social behavior phenotype. Although several studies have examined some effect of genetic variation on social behavior in *C. elegans* according to the WormBase database, very few looked at more than a limited number of genes. Therefore, it is as of yet unclear whether the limited number of genes known to be related to social behavior in the WormBase database is the result of a dearth of research (or a dearth of research included in the Wormbase database) or actually an indication that the *C. elegans* social behavior is regulated by a limited number of genes. Another issue with the comparison between human genes associated with a phenotype through GWAS and *C. elegans* genes associated with a phenotype through high-throughput phenotypic screening is that the latter mostly only finds genetic variation with large effects, while the former mostly results in findings regarding alleles with small effects. Potentially, the genetic variation previously associated with social behavior in *C. elegans* in such studies cannot be found in most human studies of social behavior because the phenotype is too severe (either to be viable or to be included in population studies such as GWAS). More research into social behavior in *C. elegans* is required to examine the genetic basis of social behavior in *C. elegans* and to determine the presence and extent of a common genetic basis for social behavior among humans and *C. elegans*. Based on these limitations, our analyses cannot be considered to provide evidence regarding the level of functional conservation in *C. elegans* orthologs of human sociability-linked genes.

Also, while we did attempt to create fair comparisons by creating random sets of protein coding genes, this method still has limitations which may have affected the results. For example, ten sets of 69 might be too low a number to get an accurate view of the level of conservation considering the size of the human protein coding genome. However, manual scoring of all human genes with *C. elegans* homologs based on our operationalization would have been untenable. This could potentially be addressed in the future by comparing genetic conservation data based on common operationalizations used as in a database of homologs of the total human protein coding genome.

## Supplementary Information

Below is the link to the electronic supplementary material.

Supplementary file1 (PDF 1151 KB)

Supplementary file2 (XLSX 33 KB)

## Data Availability

All data used for this article are available in the supplementary materials. Code used to generate the results is available upon request.
