# Supplementary material for: Assessing genetic conservation of human sociability-linked genes in C. elegans
Source: Behav Genet. 2025 Feb 21;55(2):141–52. doi: 10.1007/s10519-025-10216-2 (PMC11882721; doi:10.1007/s10519-025-10216-2)
Supplement: Supplementary file 1 — Supplementary file1 (PDF 1151 KB) [file 10519_2025_10216_MOESM1_ESM.pdf]

Supplementary Figure 1
Title: Assessing genetic conservation of human sociability-linked genes in *C. elegans*
Authors: M. C. Roozen[1] and M. J. H. Kas[1*]
[1] Groningen Institute for Evolutionary Life Sciences, University of Groningen, Groningen, the Netherlands
*Corresponding author. E-mail address: m.j.h.kas@rug.nl.

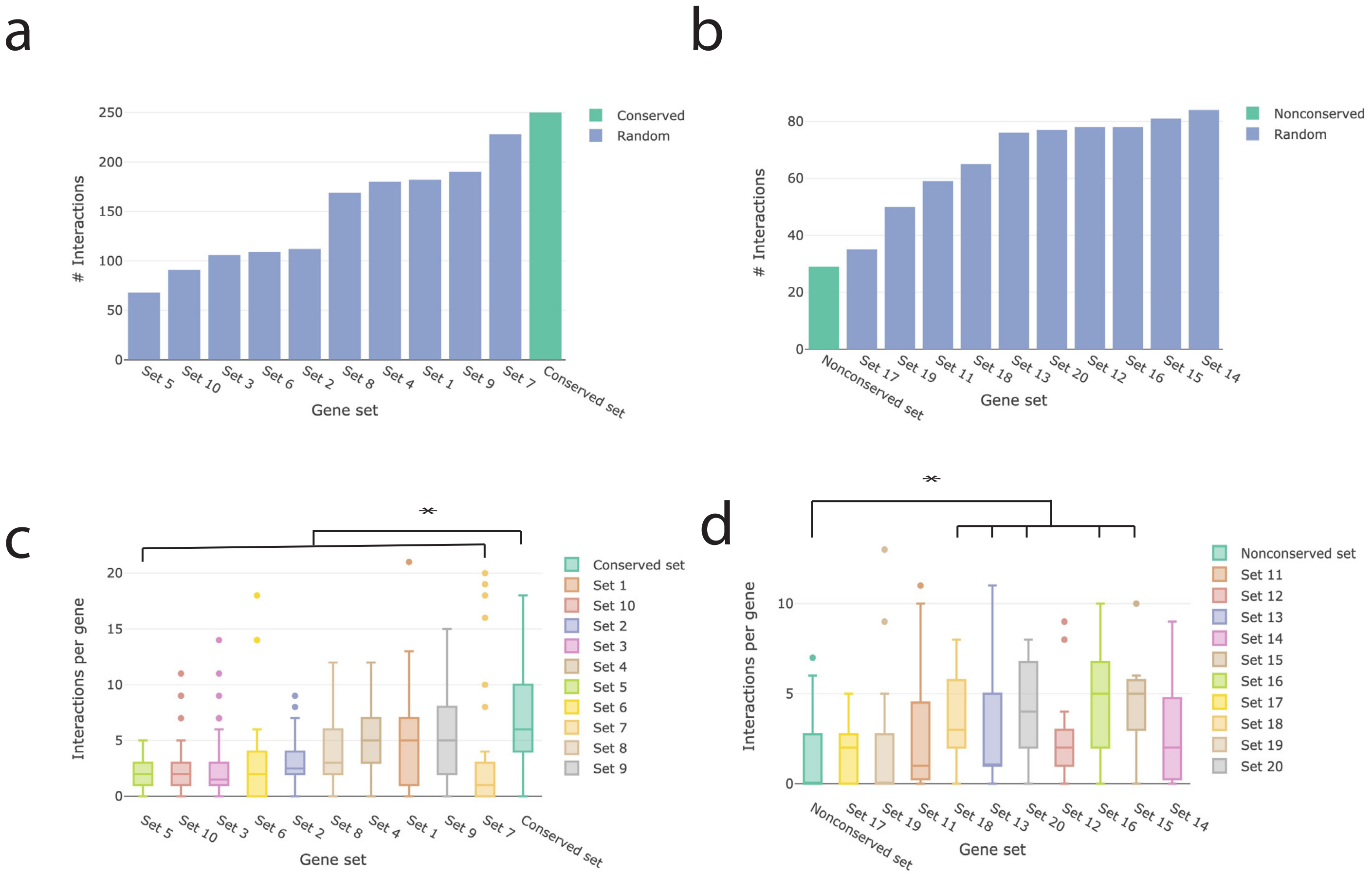

Supplementary figure 1. Interactivity between conserved sociability-linked genes, nonconserved sociability-linked genes and random gene sets allowing 20 resultant genes to be included in GeneMania. a) Total interactions between conserved sociability-linked genes and random sets of 50 human genes; b) Total interactions between nonconserved sociability-linked genes and random sets of 19 human genes; c) Interactions per gene for conserved sociability-linked genes and random sets of 50 human genes; d) Interactions per gene for nonconserved sociability-linked genes and random sets of 19 human genes.
